# How effective are entomopathogenic nematodes for vine weevil (Coleoptera: Curculionidae) biological control? A meta‐analysis

**DOI:** 10.1002/ps.70461

**Published:** 2025-12-23

**Authors:** Joe M. Roberts, Marco Corradi, Ben J. Clunie, Eugenia Fezza, W. Edwin Harris, Tom W. Pope

**Affiliations:** ^1^ Entomology Group, Centre for Crop and Environmental Sciences, Agriculture and Environment Department Harper Adams University Newport UK; ^2^ Centre for Agricultural Data Science, Agriculture and Environment Department Harper Adams University Newport UK

**Keywords:** entomopathogenic nematodes, *Otiorhynchus sulcatus*, biological control, soil temperature, protected cropping, meta‐analysis

## Abstract

**BACKGROUND:**

Vine weevil (*Otiorhynchus sulcatus*) is a globally important pest of soft fruit and ornamental crops with larvae causing significant root damage. Entomopathogenic nematodes (EPNs) have emerged as a key biological control option for larvae following restrictions on synthetic chemical insecticides, but their reported efficacy varies considerably across studies. This variability has created uncertainty about EPN effectiveness and optimal deployment strategies, limiting evidence‐based recommendations for growers.

**RESULTS:**

Across 162 comparisons from 23 studies, EPN applications significantly reduced vine weevil larval survival compared to untreated controls (Hedges' *g* = −1.60, 95% CI −1.85 to −1.36), equivalent to ≈63% fewer live larvae. All five EPN species tested (*Steinernema carpocapsae*, *S. feltiae*, *S. kraussei*, *Heterorhabditis bacteriophora*, *H. megidis*) were effective, with no significant differences among species. However, between‐study heterogeneity was high (*I* ^2^ ≈ 97%), indicating variability in outcomes despite strong average benefits. Univariate analyses identified soil temperature as the strongest moderator, with warmer temperatures (18–30 °C) associated with greater EPN efficacy. Protected cropping environments (glasshouses) also enhanced performance compared to outdoor applications. Application method (drench *versus* drip irrigation) and growing medium type showed no significant effects. However, when accounting for clustering of effects within studies using multilevel models with cluster‐robust inference, these moderator effects were reduced and no longer statistically robust. Sensitivity analyses confirmed the overall efficacy estimate was robust to study quality concerns and potential publication bias.

**CONCLUSION:**

EPNs provide reliable biological control of vine weevil larvae under field and semi‐field conditions, with effectiveness enhanced by warmer soil temperatures and protected growing environments. Although average effects are large and consistent, practitioners should expect considerable variability in outcomes and prioritise applications during warm conditions in protected environments where feasible. A lack of robust differences among species suggests that selection can be based on practical considerations such as cost and availability. © 2025 The Author(s). *Pest Management Science* published by John Wiley & Sons Ltd on behalf of Society of Chemical Industry.

## Graphical abstract

Entomopathogenic nematodes provide reliable biological control of vine weevil larvae, reducing populations by 63% on average. All five tested species are effective, with temperature (18–30 °C) being the strongest predictor of success.

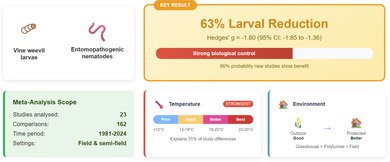

## 1 INTRODUCTION

Vine weevil, *Otiorhynchus sulcatus* F. (Coleoptera: Curculionidae), is a beetle species endemic to Europe. Once considered a sporadic glasshouse pest in this region, plant trade means that it is now an important pest of soft‐fruit and ornamental crops globally, with all life‐stages feeding on plants from a wide range of taxonomic families.^1^, ^2^, ^3^, ^4^ Greater use of polythene tunnels and glasshouses in horticultural production systems has further compounded this issue.^5^, ^6^ Protected cropping, in combination with enhanced cultivation methods such as plastic mulches, enables growers to significantly improve crop yields and extend their growing seasons.^7^ Such advancements, however, have unintended consequences as they create microclimates within a crop that confer protection against unfavourable environmental conditions and promote increased pest establishment and proliferation within crop production systems.^1^, ^8^

Adult vine weevils are nocturnal and feed on aboveground plant tissues, producing characteristic notches along leaf margins.^1^ Although this damage is usually negligible in terms of plant health, leaf notching negatively impacts plant aesthetics and reduces the economic value of ornamental crops.^1^, ^9^ Larvae hatch from eggs oviposited into the soil or growing media, feeding on belowground plant tissues such as plant roots, corms and rhizomes as they develop.^1^ Larval feeding often reduces plant vigour and, if damage is severe enough, causes total plant death. For many years larval control has relied on using various synthetic chemical insecticides such as organochlorines, organophosphates and neonicotinoids.^9^, ^10^, ^11^, ^12^ Many of these control options have, however, been withdrawn from commercial sale across Europe as a consequence of to concerns over their impact on human and environmental health.^13^, ^14^, ^15^ Synthetic chemical insecticide applications have, consequently, been superseded by biological control using entomopathogenic fungi (EPF) and entomopathogenic nematodes (EPN).^16^, ^17^, ^18^, ^19^

Two nematode genera have been shown to contain entomopathogenic species that infect vine weevil larvae: *Heterorhabditis* (Rhabditida: Heterorhabditidae) and *Steinernema* (Rhabditida: Steinernematidae).^20^ Several species from these genera are mass‐produced using bioreactor‐based liquid media *in vitro* processes and applied as drenches using conventional insecticide application equipment or through dripline irrigation systems.^21^, ^22^ Infective juveniles (3^rd^‐instar EPNs) are applied to crops where they actively seek or ambush their insect hosts and gain entry through cuticle openings such as the mouth or spiracles, releasing bacteria that proliferate and cause death by septicaemia within 48 h.^23^, ^24^, ^25^, ^26^ Most research on using EPNs for vine weevil larvae control in soft fruit and ornamental crops has focused on five species (Table 1). Each of these EPN species is thought to differ in their efficacy with conflicting evidence as to which offers the most effective control.^27^, ^28^ It is likely that this uncertainty is, in part, a result of the influence of environmental factors as well as application technique or plant growing media.^29^ A better understanding of the importance such factors have in determining EPN efficacy for vine weevil larval control would be beneficial to growers. To help address this uncertainty, a meta‐analysis was carried out to compare the efficacy of five EPN species against vine weevil larvae and determine which factors influence control success. The specific factors examined were: (1) EPN species to reflect differences in foraging strategies; (2) crop type; (3) soil/substrate temperature as this modulates EPN activity; (4) application method as this has an impact on EPN distribution; and (5) growing context as this can influence nematode survival.

**Table 1:** Entomopathogenic nematode (EPN) species considered within the meta‐analysis. Adapted from Bennison *et al.* ^66^

EPN species	Optimal application temperature (°C)	Bacterial symbiont	Foraging strategy
*Heterorhabditis bacteriophora* (Poinar, 1976)	12–30	*Photorhabdus* spp.	Cruiser
*Heterorhabditis megidis* (Poinar, 1987)	>10	*Photorhabdus* spp.	Cruiser
*Steinernema carpocapsae* (Weiser, 1955)	>10	*Xenorhabdus* spp.	Ambusher
*Steinernema feltiae* (Filipjev, 1934)	8–33	*Xenorhabdus* spp.	Intermediate
*Steinernema kraussei* (Steiner, 1923)	5–30	*Xenorhabdus* spp.	Cruiser

## 2 METHODS

### 2.1 Literature survey strategy

Science Direct, CAB Direct and Web of Science were searched to generate a database of articles investigating the efficacy of the five selected EPN species for vine weevil control in outdoor, polytunnel‐ or glasshouse‐grown soft fruit and ornamental crops (Table 1). To identify literature on this topic, the following search terms were used in each search engine: (‘vine weevil*’ OR ‘Otiorhynchus sulcatus’) AND (nematode* OR Steinernema OR carpocapsae OR feltiae OR kraussei OR Heterorhabditis OR megidis OR bacteriophora). Grey literature identified through CAB Direct was included to reduce publication bias risk. Studies were searched between January 1981, when EPNs were first produced for commercial biological control purposes and December 2024 with no geographical limit.^30^ For a study to be included in the meta‐analysis it was screened to determine whether it met the following five criteria: (1) written in English language; (2) targeted vine weevil larvae with adult studies excluded; (3) used one or more of the five selected EPN species; (4) focused on soft fruit or ornamental crops; and (5) completed either outdoors, or within a polythene tunnel or glasshouse. Laboratory experiments and anecdotal observations were excluded to avoid inclusion of unrealistic or unquantifiable data. Each study was screened for data availability with only those reporting mean numbers of live larvae in treated and control groups together with a variance estimate [standard deviation (SD) or standard error (SE)] and sample size retained. This process yielded 162 experimental comparisons from 23 studies.

### 2.2 Study quality and risk classification

The mean total sample size (treatment + control) for each study was calculated to assess potential risk of bias. Studies were classified as low risk (mean total sample size ≥30), moderate risk (15–29) or high risk (<15). This classification recognises that larger sample sizes provide more precise effect estimates. High risk studies were excluded in sensitivity analyses.

### 2.3 Risk of bias assessment

Risk of bias (RoB) was assessed using a simple domain‐based approach adapted for field/semi‐field trials. The following domains were judged at the study or comparison level where reported: randomisation of experimental units, allocation/concealment or comparability of units, blinding of outcome assessment, outcome measurement/reporting and other potential sources of bias. Each domain was coded as low RoB, some concerns or high RoB with terms such as ‘unclear’ normalised to some concerns; unreported items were left missing. Where only study‐level RoB was available, the judgement was applied to all comparisons from that study. RoB was not used to weight studies in the primary meta‐analysis; instead, it informed sensitivity and moderator analyses, by (i) exclusion of any comparison flagged high in any domain (‘any‐high’) and (ii) incorporating RoB‐as‐moderator models, including multilevel specifications with CR2 small‐sample cluster‐robust tests.

### 2.4 Data extraction and effect size calculation

The mean number of live vine weevil larvae in the EPN treatment (${\bar{X}}_{\mathrm{EPN}}$) and control (${\bar{X}}_{\text{Control}}$) groups along with their corresponding SD or SE and sample sizes ($n_{\mathrm{EPN}}$ and $n_{\text{Control}}$) were extracted from eligible comparisons (treatment–control pair) in each study. When data were only available from figures, webplotdigitizer was used to extract the numeric values.^31^ If a study reported SE instead of SD then it was converted using SE × √*n*. Comparisons lacking any measure of variance or having reported an SD of zero were excluded as an effect size cannot be computed without a non‐zero variance estimate. Outcomes were standardised as the mean number of live larvae remaining so that smaller values indicate greater efficacy (i.e. higher pest mortality resulting from the EPN treatment). The treatment effect for each comparison was quantified using a bias‐corrected standardised mean difference in the form of Hedges' *g*.^32^ This was calculated using:

$$g=J\times \frac{{\bar{X}}_{\mathrm{EPN}}-{\bar{X}}_{\text{Control}}}{SD_{\text{pooled}}}$$

where

$$SD_{\text{pooled}}=\sqrt{\frac{\left(n_{\mathrm{EPN}}-1\right)SD_{\mathrm{EPN}}^{2}+\left(n_{\text{Control}}-1\right)SD_{\text{Control}}^{2}}{n_{\mathrm{EPN}}+n_{\text{Control}}-2}}$$

and

$$J=1-\frac{3}{4\left(n_{\mathrm{EPN}}+n_{\text{Control}}\right)-9}$$

Negative *g* values indicate fewer live larvae in the treated group than in the control, reflecting a reduction in vine weevil survival resulting from EPN treatment. Sampling variance for *g* was calculated using unbiased formulas (metafor vtype = ‘UB’) that account for each comparison's sample sizes.^33^ When a study reported multiple comparisons (e.g. several EPN treatments or conditions against a shared control), a mean effect size was not calculated as each comparison was treated as a separate data point, retaining the clustering of effects within studies for the meta‐analysis model. As a scale‐sensitivity analysis, the log response ratio (ln ROM) with Hedges–Olkin variance (‘HO’) was used to apply a small continuity offset when nonpositive means occurred and interpreted exp(ln ROM) as the proportional change in live larvae.

### 2.5 Statistical analysis

All analyses were carried out in R v4.4.0 using the metafor package for meta‐analysis in combination with meta for supplementary calculations, clubsandwich for variance estimation and ggplot2 for data visualisation.^34^, ^35^, ^36^, ^37^, ^38^ The primary meta‐analysis used a random‐effects model fitted by restricted maximum likelihood (REML) to estimate the overall mean effect size. A random‐effects model was selected because heterogeneity was expected owing to varying experimental conditions. This model yields an estimate of the average treatment effect while assuming that the true effects are distributed around that mean rather than identical across studies. Between‐study heterogeneity was quantified using Cochran's *Q* test, between‐study variance (τ^2^) and percentage of variability attributable to heterogeneity (*I* ^2^). A 95% prediction interval (PI) is reported for the distribution of true effects and, for interpretability, the probability that a new study favours treatment, –(θ <0), under the fitted random‐effects distribution.

Six *a priori* moderator analyses were carried out to explore potential sources of heterogeneity: EPN species (five species); crop type (soft‐fruit *versus* ornamental); cropping context (open‐field *versus* polytunnel *versus* glasshouse); application method (standard drench *versus* drip irrigation); growing medium (peat‐based, coir, bark‐containing, soil); and soil temperature at application (categorised into ranges). Each moderator was tested in a separate analysis using a mixed‐effects meta‐regression under a random‐effects structure. In these models, the moderator was included as a fixed effect to assess differences in mean Hedges' *g* between subgroups. Omnibus between‐level differences were tested using likelihood‐ratio tests (LRT) fitted under maximum likelihood (ML), whereas subgroup estimates and heterogeneity metrics were obtained under REML. A moderator effect was considered statistically significant if the omnibus test (QM) for between‐group differences was significant or if the 95% CI for the difference did not include zero. For each moderator, pseudo‐*R* ^2^ was calculated as the proportional reduction in τ^2^ under REML relative to the baseline model and truncated at 0 to avoid negative values. For two‐level moderators, the subgroup contrast was additionally evaluated with a Wald test. In addition to the separate univariate moderator tests, a multivariable meta‐regression was carried out that included all six moderators simultaneously. This facilitated identification of which factors retained independent explanatory power when controlling for the others. A conventional random‐effects meta‐regression and a multilevel meta‐regression with random intercepts for each study ID and effects nested within studies also were fitted with inference for the multilevel model using small‐sample cluster‐robust tests (CR2) with Satterthwaite degrees of freedom.

Sensitivity analysis was carried out to assess the robustness of the meta‐analysis. First, the meta‐analysis was repeated after excluding high‐risk studies as defined by the sample size criteria above to evaluate whether smaller experiments unduly influenced the results. Second, a leave‐one‐study‐out analysis was carried out to sequentially remove each study and recalculate the overall Hedges' *g* to check for any study that disproportionately affected the pooled outcome. Third, the potential nonindependence of effect sizes drawn from the same study was addressed by fitting a two‐level multilevel meta‐analysis model with random intercepts for each study and effects nested within studies. This multilevel model partitioned the variance into between‐ and within‐study components and accounts for the correlation among effect sizes from the same study. Additionally, a cluster‐robust variance estimator was applied to obtain hypothesis tests and confidence intervals that remain valid even if there are dependencies among effects or other violations of standard meta‐analysis assumptions. The CR2 estimator was implemented in clubSandwich to obtain small‐sample–adjusted tests. This approach provides an extra layer of confirmation that inference (e.g. significance of the overall effect and moderators) is not an artifact of treating multiple comparisons from one study as independent observations.^37^ Publication bias was assessed visually using contour‐enhanced funnel plots and statistically using Egger's regression test and the Begg–Mazumdar rank‐correlation test, whereas trim‐and‐fill was applied to estimate the number of missing studies and adjust the pooled effect if necessary.^39^, ^40^ Where domain‐level risk‐of‐bias judgements were available, sensitivity analyses excluding any comparison flagged ‘high’ in any domain were carried out and fitted RoB‐as‐moderator models with CR2‐robust inference.

Moderator variables were not completely observed across all comparisons. Complete‐case (listwise) deletion analysis was used for each moderator, where comparisons with missing data for a specific moderator were excluded only from that moderator's analysis while being retained for the overall pooled effect and other moderator analyses. Imputation was not used because: (1) missingness was relatively low for most moderators; (2) imputation would introduce additional assumptions about the missing data mechanism; and (3) complete‐case analysis is standard practice in ecological meta‐analysis where moderator availability often varies by study design (REF).^41^, ^42^ To assess whether missing data biased the overall effect estimate, a sensitivity analysis comparing the pooled effect using all available data *versus* only those comparisons with complete information across all six moderators was carried out. Little's test of Missing Completely At Random (MCAR) was carried out using naniar to evaluate whether the missing data followed a systematic pattern.^41^, ^43^, ^44^

## 3 RESULTS

### 3.1 Literature search

**Figure 1:**
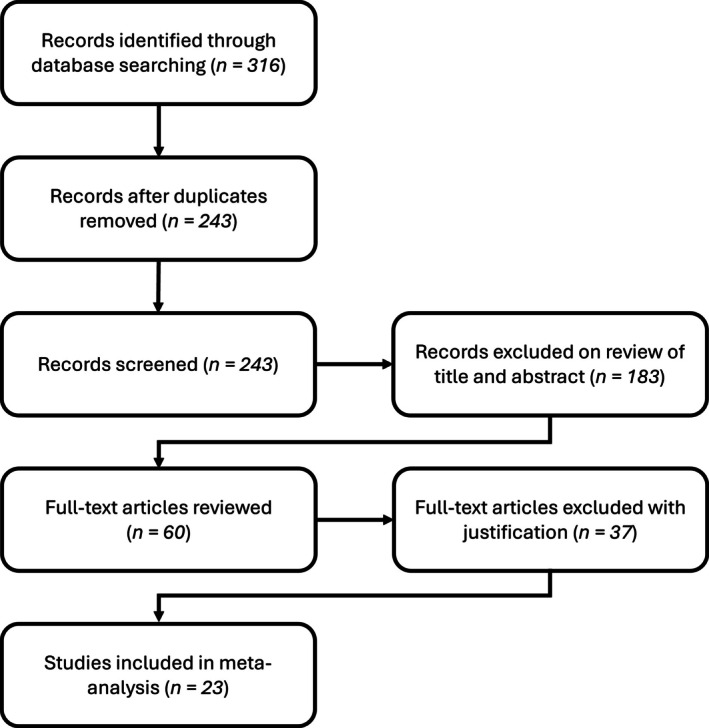
Literature search and study‐selection flow diagram. The diagram traces the PRISMA‐style screening process from 316 records initially retrieved, through deduplication (243), title/abstract screening (183 exclusions), full‐text assessment (60 articles reviewed; 37 exclusions with reasons), to the 23 studies retained for quantitative synthesis.

The literature search identified 316 candidate publications, of which 23 met all inclusion criteria for the meta‐analysis (Fig. 1). These 23 studies yielded a total of 162 independent experimental comparisons examining EPN efficacy against vine weevil larvae. Most included studies were completed in Europe (*n* = 13; 54%), with the largest contributions from the UK and Ireland (*n* = 9; 39%) and the United States (*n* = 8; 35%) with the remaining studies (*n* = 2; 11%) from New Zealand, reflecting a highly conservative geographic range within the meta‐analysis. All experiments were completed under field or semi‐field conditions (open‐field, polytunnel or glasshouse) on soft‐fruit or ornamental crops with each providing the necessary data (treatment and control means with variance measures) for effect size calculation.

### 3.2 Overall efficacy of EPNs against vine weevil larvae

EPN treatments produced a highly significant reduction in vine weevil larval survival relative to untreated controls across all 162 comparisons. Across all 162 comparisons, EPN treatments produced a large and highly significant reduction in vine weevil larval survival (standardised effect size *g* = −1.60, 95% confidence interval: −1.85 to −1.36; *P* < 0.001). The magnitude of this effect varied considerably among studies (*I* ^2^ = 97.2%), with most variation reflecting differences in experimental conditions rather than chance alone. Individual moderators explained some of this variation (temperature: 31%; cropping context: 9%; growing medium: 7%), whereas the combined model including all moderators explained 32% of the total variation. The individual moderator analyses explained substantial proportions of this heterogeneity (temperature: *R* ^2^ = 30.8%; context: *R* ^2^ = 9.4% and Substrate: *R* ^2^ = 7.2%), whereas the multivariable model combining all moderators accounted for 32% of between‐study variance. Any remaining unexplained variance likely reflects unmeasured factors (e.g. EPN application rate, soil moisture, EPN strain virulence) and natural biological variation. Although the average effect across all studies was strongly negative (favouring EPN treatment), individual study outcomes ranged from very large reductions in larval survival to occasional small increases, indicating that results can be context‐dependent. Approximately 86% of future studies would be expected to show a beneficial effect of EPN treatment. Because some studies contributed multiple comparisons, the data also were analyzed using a statistical model that accounts for the nonindependence of multiple measurements from the same experiment. This more conservative approach yielded a slightly smaller but still large overall effect (*g* ≈ −1.45; 95% CI –1.96 to −0.95), confirming that the treatment benefit is robust to different analytical approaches. The evidence for a large overall EPN treatment effect is robust to assumptions about effect size independence. Expressing results as a percentage rather than standardised units, EPN treatment resulted in ≈63% fewer live larvae on average compared to untreated controls.

### 3.3 Entomopathogenic nematode species

**Figure 2:**
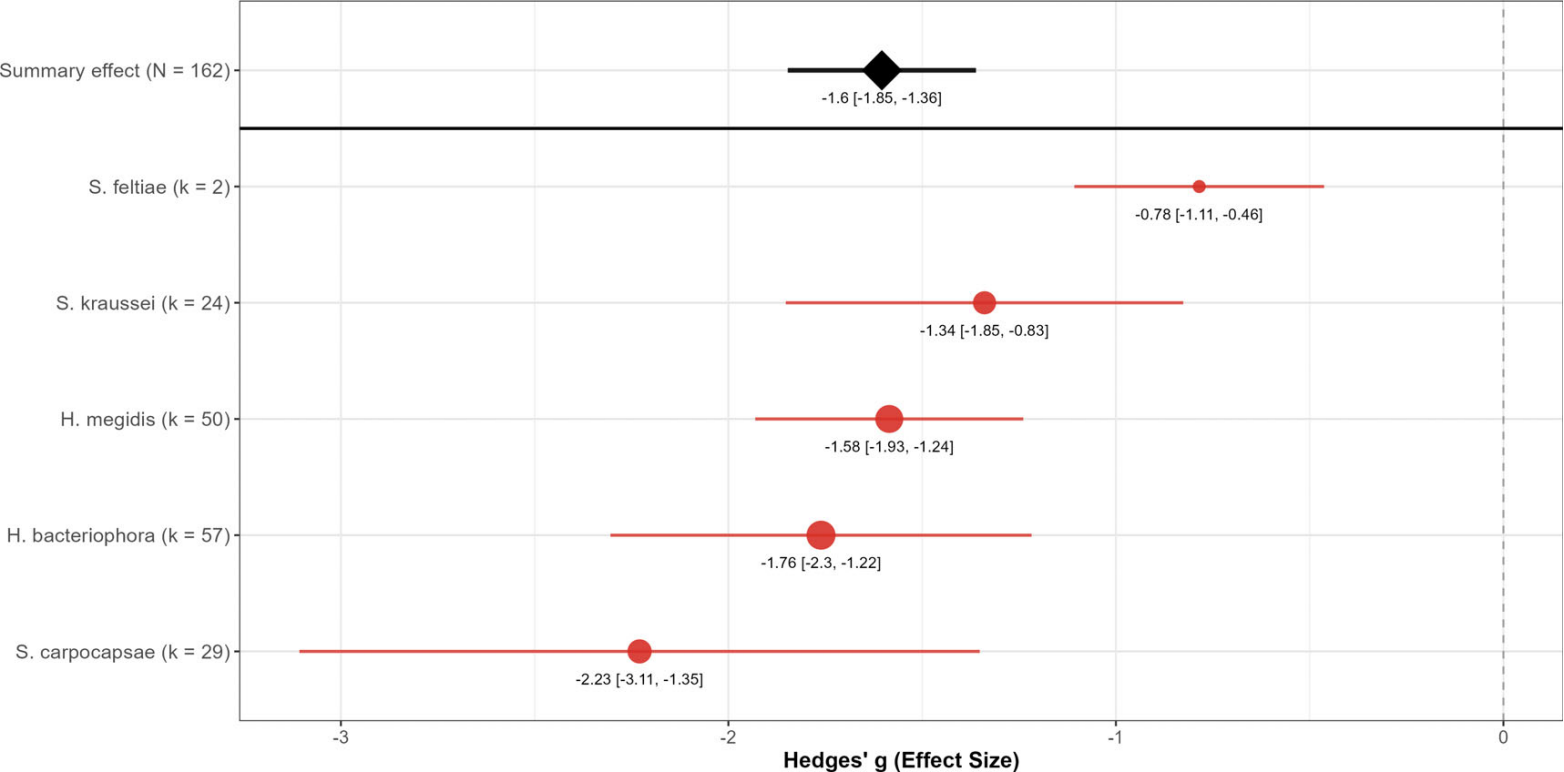
Species‐specific EPN efficacy against vine weevil larvae. Point estimates (filled circles) and 95% confidence intervals (red bars) show Hedges' g for each EPN species; k values in parentheses after each species label indicate the number of comparisons contributing to that subgroup. Circle size is proportional to k. The black diamond (with CI) gives the overall random effects mean across all 162 comparisons; the dashed line marks the null (g = 0).

All five EPN species evaluated in this meta‐analysis significantly reduced vine weevil larval survival, though their effect sizes varied in magnitude (Fig. 2). Among the five EPN species, *S. carpocapsae* and *H. bacteriophora* yielded the most pronounced mean effects (*g* > −2), whereas *S. feltiae* exhibited a more modest average effect (*g* < −1). However, differences among species were not statistically significant (*P* = 0.663), and species identity explained essentially none of the variation in outcomes among studies (*R* ^2^ < 0%). In practical terms, no single species provided ‘better’ control to the others as their confidence intervals overlapped substantially. Although there was a tendency for *S. carpocapsae* and *H. bacteriophora* to achieve slightly greater larval suppression and for *S. feltiae* to be less effective, these distinctions were not robust given the variability among studies.

### 3.4 Crop type and cropping context

**Figure 3:**
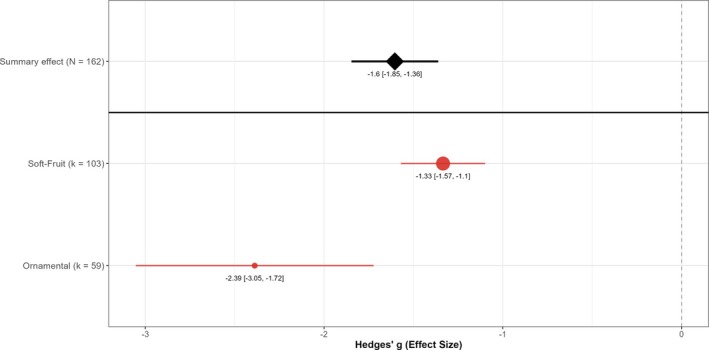
Influence of crop type on EPN efficacy against vine weevil larvae. Point estimates (filled circles) and 95% confidence intervals (red bars) show Hedges' g for soft‐fruit (k = 103 comparisons) and ornamental crops (k = 59). Circle size is proportional to k. The black diamond (with CI) gives the overall random effects mean across all 162 comparisons; the dashed line marks the null (g = 0).

**Figure 4:**
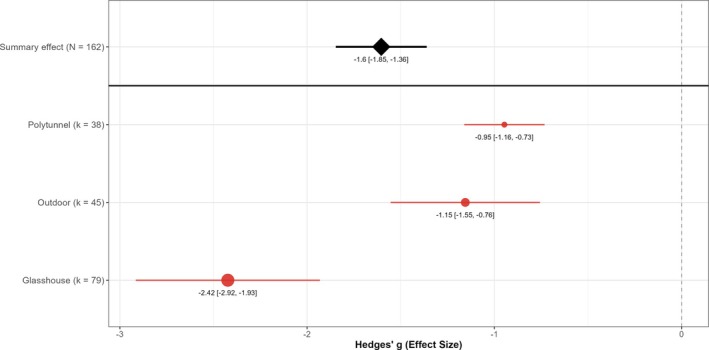
Influence of cropping context on EPN efficacy against vine weevil larvae. Point estimates (filled circles) and 95% confidence intervals (red bars) show Hedges' *g* for glasshouse (*k* = 79 comparisons), outdoor (*k* = 45) and polytunnel experiments (*k* = 38). Circle size is proportional to *k*. The black diamond (with CI) gives the overall random effects mean across all 162 comparisons; the dashed line marks the null (*g* = 0).

EPN applications were effective in reducing vine weevil larvae in both soft‐fruit and ornamental crops (Fig. 3). Although ornamental crops showed statistically larger effects than soft‐fruit crops (*P* = 0.006), crop type explained almost none of the variation among studies (*R* ^2^ ≈ 0%). Given the substantial overlap in confidence intervals and minimal explanatory power, this difference has limited practical significance for growers choosing between crop types. Cropping context, by contrast, significantly moderated EPN efficacy (Fig. 4). The differences among cropping contexts were highly significant (*P* < 0.001), with context explaining ≈9% of the variation among studies. Glasshouse applications achieved significantly greater effects than either outdoor or polytunnel applications (pairwise *P* < 0.01 for both comparisons), whereas outdoor and polytunnel settings did not differ significantly from each other (*P* = 0.58). This indicates that EPNs are most efficacious when applied under protected glasshouse conditions, with relatively smaller but still negative effects observed in outdoor and polytunnel environments.

### 3.5 Application method and growing medium

**Figure 5:**
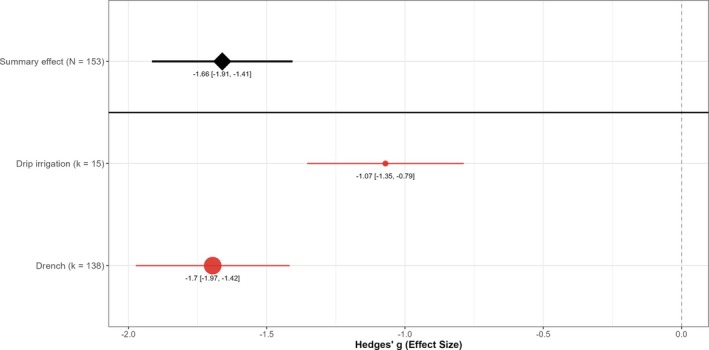
Effect of application method on EPN efficacy against vine weevil larvae. Hedges' *g* values (filled circles) with 95% confidence intervals (red bars) are shown for drench applications (*k* = 138 comparisons) and drip irrigation (*k* = 15). Circle size is proportional to *k*. The black diamond and horizontal line show the overall random effects mean across 153 comparisons; the dashed line marks the null (*g* = 0).

**Figure 6:**
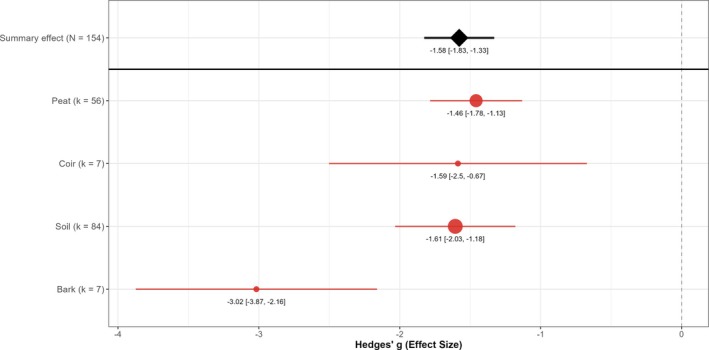
Effect of growing‐medium type on EPN suppression of vine weevil larvae. Points (filled circles) show Hedges' *g* for peat (*k* = 56 comparisons), coir (*k* = 7), soil (*k* = 84) and bark substrates (*k* = 7); horizontal red bars are 95% confidence intervals and circle diameter scales to *k*. The black diamond (with CI) gives the overall random effects mean across 154 comparisons, while the dashed line marks the null (*g* = 0).

Application method did not significantly impact EPN efficacy against vine weevil larvae (Fig. 5). Although drip irrigation showed a slightly smaller effect than drench applications, this difference was not statistically significant (*P* = 0.58), and application method explained none of the variation among studies (*R* ^2^ ≈ 0%). Given the relatively small number of drip irrigation experiments (15%), confidence intervals were wide, preventing definitive conclusions about method superiority. Both approaches resulted in large larval reductions with overlapping efficacy estimates, suggesting that practical considerations such as cost and infrastructure compatibility should guide method selection. Growing medium showed a marginally nonsignificant trend toward differential efficacy (*P* = 0.058), explaining ≈7% of the variation among studies (Fig. 6). Bark‐containing media produced the largest descriptive effect (g ≈ −3.0) and coir showed slightly smaller effects on average, yet confidence intervals overlapped substantially across all substrates. No growing medium type was statistically distinguishable from others in terms of EPN efficacy. However, the relatively small sample sizes for coir and bark substrates (*k* = 7) contributed to wider uncertainty around these estimates. Within the range of common horticultural growing media tested, there was no evidence that growing media consistently influences the degree of vine weevil control achieved by EPNs.

### 3.6 Effect of soil temperature

**Figure 7:**
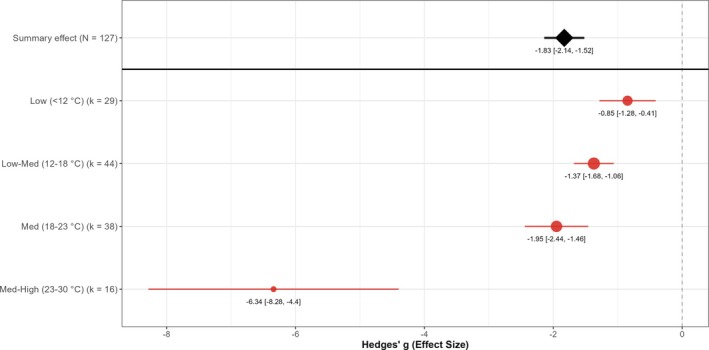
Influence of soil temperature on EPN efficacy against vine weevil larvae. Hedges' *g* estimates (red circles) with 95% confidence intervals are shown for four temperature categories: Low (<12 °C, *k* = 29), Low–Medium (12–18 °C, *k* = 44), Medium (18–23 °C, *k* = 38) and Medium‐High (23–30 °C, *k* = 16). Circle diameter is proportional to the number of comparisons (*k*). The black diamond indicates the overall random effects mean across 127 comparisons; the dashed line marks the null (*g* = 0).

Soil temperature at application was the strongest factor influencing EPN efficacy (*P* < 0.001), explaining ≈31% of the variation among studies (Fig. 7). Subgroup estimates followed a clear temperature gradient, with higher soil temperatures consistently associated with greater reductions in vine weevil larval survival. The warmest category (23–30 °C) produced the largest effects, and even between adjacent temperature ranges there were notable improvements in efficacy with increased warmth. However, the difference between 12 and 18 °C and 18–23 °C was only marginally significant. At very low soil temperatures (<12 °C), nematode performance was much less reliable, whereas all EPN species demonstrated effective infection and killing of vine weevil larvae at moderate‐to‐high temperatures (≥18 °C).

### 3.7 Multivariable meta‐regression

A multivariable meta‐regression was used to examine all six moderators simultaneously and determine which factors independently contributed to heterogeneity in EPN efficacy. This included all comparisons with complete data for the moderators (*N* = 117 comparisons after excluding cases with missing moderator information). The combined model including all factors significantly improved predictions compared to the overall average alone (*P* < 0.001) and explained approximately 32% of the variation among studies. Consistent with the univariate results, soil temperature was the strongest independent predictor of EPN efficacy in the multivariable model under model‐based standard errors, and cropping context also appeared important. However, when using a more conservative statistical approach that accounts for multiple comparisons from the same studies, none of the individual factors remained statistically significant (all *P* > 0.05), suggesting that the apparent effects may be less robust than initially indicated. The intercept of the multivariable regression was significantly negative (*P* < 0.001), reinforcing that even under less favourable conditions, an EPN treatment tends to outperform an untreated control. Overall, temperature and context were the principal drivers in univariate analyses but their partial effects were not robust under multilevel CR2 inference.

### 3.8 Publication bias, sensitivity analyses and missing data

**Figure 8:**
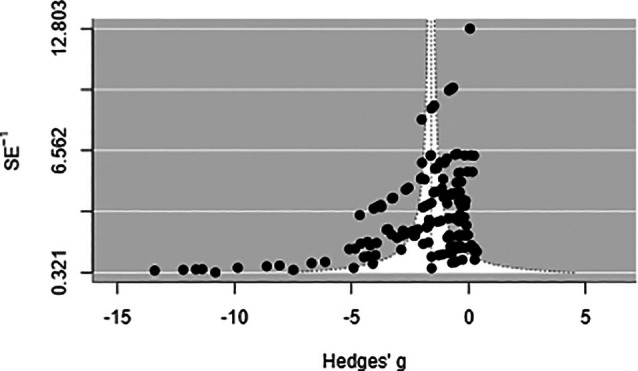
Funnel plot of the 162 Hedges' *g* estimates against their SEs. The white diagonal curves delimit the pseudo‐95% confidence triangle expected under a symmetrical (bias‐free) distribution, whereas the dashed vertical line marks the pooled random‐effects mean (*g* ≈ −1.60). The scarcity of small studies near the null and the cluster of small studies with very large negative *g* values produce the obvious left‐skew. Egger's regression confirmed significant small‐study effects (bias intercept ≈ −0.54, *P* < 0.0001). Duval & Tweedie's trim‐and‐fill procedure suggested no missing studies on the more efficacious side, and the overall mean was therefore unchanged.

Visual inspection of effect sizes plotted against study precision (Fig. 8) identified asymmetry, with small studies tending to report larger treatment benefits than larger studies. Statistical tests confirmed this pattern (Egger's test: *P* < 0.001), suggesting potential small‐study bias. However, a trim‐and‐fill procedure designed to estimate and adjust for potentially missing studies did not identify any absent studies that would change the pooled effect estimate. When the 10 comparisons rated as high risk of bias in any domain were excluded, the pooled effect was essentially unchanged (*g* = −1.55 *versus* −1.60 in the full dataset), indicating that study quality did not substantially influence the overall conclusion. Likewise, RoB ratings were not associated with effect size when accounting for clustering of comparisons within studies (*P* = 0.333). Although some evidence of small‐study effects exists, sensitivity analyses suggest minimal impact on the central finding that EPNs reduce vine weevil larval survival. Sensitivity analyses were carried out to assess the robustness of the meta‐analysis findings. First, the meta‐analysis was repeated after excluding high‐risk studies. Removing these smaller, potentially less reliable experiments reduced the dataset to *k* = 105 comparisons. The overall effect in this restricted dataset was nearly identical to the full analysis (*g* = −1.56 *versus* −1.60), indicating that smaller studies were not inflating the efficacy estimate. Second, a leave‐one‐out analysis confirmed that no single study unduly influenced the meta‐analysis outcome. Sequentially removing each of the 23 studies produced pooled estimates that varied modestly around the overall mean and all remained highly significant. Therefore, the conclusion that EPN treatments substantially reduce vine weevil larvae is not driven by any outlier study or any specific low‐quality experiment. Finally, statistical models that accounted for multiple comparisons from the same studies confirmed the overall treatment effect but indicated that individual moderator effects were less robust than suggested by simpler analyses.

Missing data within the moderators ranged from 0% (species, crop, context) to 25% (temperature). Little's MCAR test indicated some systematic pattern to missingness (χ^2^ = 17.28, df = 7, *P* = 0.016), primarily reflecting that temperature data were less frequently reported in outdoor field studies compared to controlled glasshouse experiments. To assess potential bias, the overall pooled effect using all data [*k* = 162, *g* = −1.60 (95% CI: −1.85, −1.36)] *versus* only comparisons with complete moderator data [*k* = 117, *g* = −1.83 (−2.15, −1.50)] was compared. The complete‐case estimate (*g*) was 0.23 more negative than the overall pooled effect, suggesting that including comparisons with partial moderator data yielded a slightly more conservative overall effect estimate.

## 4 DISCUSSION

This meta‐analysis confirms that EPNs exert a suppressive effect on vine weevil larvae under a wide range of conditions. In experiments with soil temperatures ≥12 °C, across both protected and outdoor crops using both drench and drip line application methods, EPN treatments reduced larval populations relative to untreated controls. On the ratio scale, this corresponded to ≈63% fewer live larvae on average, representing substantial biological control efficacy. This finding reinforces the view that EPNs are an effective and reliable biological control option for vine weevil larvae.^45^, ^46^ The overall efficacy observed in this present study was robust to sensitivity analyses as when higher‐risk or outlier studies were excluded there was a negligible impact on the pooled effect size. Thus, the conclusion that EPNs substantially reduce vine weevil larvae is supported by a convergence of evidence and is not driven by any single study or biased subset of data. There was, however, considerable heterogeneity in outcomes among individual studies. Consistent with this, although the average effect was strongly positive, individual study outcomes ranged widely from very large reductions to occasional small increases in larval survival, and ≈86% of future studies would be expected to show beneficial effects. The susceptibility of vine weevil larvae to EPN infection is well established, yet EPN efficacy can be impacted by differences in environmental conditions and methodologies.^20^, ^47^ The results from this meta‐analysis align with these previously described patterns, as several moderators of EPN efficacy were identified that help explain why some experiments report better vine weevil control than others.

All five EPN species evaluated in this meta‐analysis significantly reduced vine weevil larval populations, although differences in performance among species were relatively modest, with *S. carpocapsae* and *H. bacteriophora* producing the largest mean larval reductions followed by *H. megidis*, *S. kraussei* and *S. feltiae*. This ranking aligns with the comparative results of Ansari *et al*., who also found *S. carpocapsae* and *H. bacteriophora* to be most effective against vine weevil larvae. However, *S. carpocapsae* showed the most variable outcomes among species, with performance ranging from excellent to modest across different studies. Ansari *et al*. also noted greater variability in *S. carpocapsae* performance.^48^ A potential explanation for the observed variability is that foraging behaviour and host‐finding strategy differ between nematode taxa in ways that impact field consistency. *Heterorhabditis* spp. and *S. kraussei* are cruiser foragers that actively move through soil to locate hosts, which may give them an advantage against relatively sedentary vine weevil larvae.^24^ By contrast, *S. carpocapsae* is an ambusher that waits for mobile hosts. Nevertheless, *S. carpocapsae* is known to produce toxic proteins via its bacterial symbiont that can kill hosts rapidly.^49^ Such traits are likely to explain why *S. carpocapsae* can still provide excellent control in many cases but may also contribute to outcome variability if environmental conditions or larval behaviour are not conducive to this nematode's ambush strategy.

Temperature was identified as a key moderator influencing EPN efficacy, explaining the greatest heterogeneity among single moderators at ~31%. Applications made when growing medium temperatures were in the ~18–30 °C range yielded the best control, with efficacy declining as temperatures dropped. The meta‐analysis supports current best practice recommendations to apply EPNs when soil temperatures are 20–25 °C for optimal pest control performance.^47^ Subgroup estimates followed a pronounced gradient, with the 23–30 °C category showing the largest effects and temperature explaining the greatest share of heterogeneity among single moderators (~31%). At low temperatures (<12 °C), EPN activity is greatly reduced for most species, and correspondingly the meta‐analysis identified much smaller effect sizes in those conditions. Field studies corroborate that cool soils can limit EPN performance. For example, Booth *et al*. observed that the efficacy of *Steinernema* spp. and *Heterorhabditis* spp. nematodes against root weevils in berry crops was most limited by cool soil temperatures typical of the Pacific Northwest Spring, although soil structure and crop features also play a role.^50^ In their experiments, springtime applications often coincided with vine weevil larvae being present in late instars or pupae when soil temperatures were marginal, leading to inconsistent control. Likewise, Georgis *et al*. reported that EPN products show low efficacy under unfavourable field conditions, such as suboptimal climate or timing, which has been one factor limiting their success in practice.^18^ There are, however, certain EPN species such as *S. kraussei* that specialise in colder conditions and provide good levels of vine weevil control.^51^ This cold‐active species has shown particular promise for applications in cooler climates or during suboptimal temperature windows, offering potential solutions when other species perform poorly.^51^ These findings stress that most EPN species perform best when applied under warm environmental conditions and when target larvae are present and active.

In general, EPNs were highly effective in both the soft fruit and ornamental crops analyzed, but effect sizes were larger and less variable in ornamentals. The crop‐type contrast was statistically significant in aggregate, although it explained essentially none of the between‐study variance (*R* ^2^ ≈ 0%), suggesting limited practical contribution to heterogeneity. One potential explanation for this variability is differences in root architecture and growth medium between these production systems. Ornamental crops in the included studies were often pot‐grown in semi‐controlled environments such as glasshouses, sometimes with simpler root systems or soilless media, whereas soft fruits often have very fibrous root networks and, in some experiments, were grown in open field soil. Complex, filamentous root systems can physically impede nematode movement and reduce the probability of host contact. Demarta *et al*. demonstrated that root architecture can decrease the foraging efficiency of EPNs and lead to lower infection rates.^52^ By contrast, many ornamental plants have more woody or tap‐rooted systems that might be easier for nematodes to navigate, or they are grown in media such as peat that allow better nematode dispersal. It is also relevant that nearly all ornamental‐crop studies in our analysis were carried out in glasshouse conditions, whereas a portion of soft‐fruit studies were outdoors. Protected cultivation in glasshouses or polytunnels generally provides more favourable and stable environmental conditions for EPN survival, whereas outdoor applications can be subject to desiccation or temperature stress.^47^ This could contribute to the higher variability observed in some field studies on soft fruit. EPNs have long been considered a proven control solution for vine weevil control in glasshouses, but achieving the same consistency in outdoor grown crops is more challenging without environmental control.^18^ The present study is consistent with these findings as EPNs always provided a benefit and cropping context emerged as a significant moderator (glasshouse > outdoor/polytunnel), explaining a modest portion of heterogeneity (~9%).

All growing media supported a large nematode effect in absolute terms, but we detected the highest average efficacy in bark‐based media and the lowest in coir‐based media. However, growing media effects did not reach conventional significance (near‐significant trend; small *k* for bark/coir), so differences should be interpreted cautiously. Bark and peat media are characterised by high porosity that retains moisture films, which are likely to enhance nematode survival and mobility. Kakouli‐Duarte *et al*. likewise found that peat‐based potting mixes improved EPN performance owing to better moisture availability.^53^ Coir, however, was associated with smaller effect sizes in the meta‐analysis. It may be that chemical and microbial differences in coir, or perhaps its structure at the micro‐scale, might negatively affect nematode persistence or host‐finding compared to other growing media.^54^, ^55^ However, relatively few studies in the dataset used coir, so the lower efficacy also could reflect limited data. As coir is increasingly used in horticulture, the apparent gap in EPN performance and the lack of focused studies in coir merit further investigation.^56^ Optimising vine weevil biological control will require understanding these three‐way interactions between EPN, host plant and growing medium.

Application method did not significantly influence efficacy in this meta‐analysis. Studies where nematodes were applied via drip irrigation through existing watering lines reported lower efficacy than those using a one‐time drench application to the growing medium. Given the relatively small number of drip trials and wide CIs, mechanistic arguments for drip (e.g. repeated, even delivery) remain plausible but unproven; both methods achieved large average reductions, so practical considerations should guide choice. Drip irrigation is likely to distribute nematodes more evenly and repeatedly into the soil profile, maintaining a higher concentration of infective juveniles around the plant roots over time. This method essentially integrates nematodes into routine watering, which may lead to better coverage of the target pest's habitat. Kakouli‐Duarte *et al*. provided early evidence that drip systems can effectively deliver EPNs and control vine weevil larvae.^53^ In the meta‐analysis, drip‐applied nematodes had slightly more variable outcomes than drenches, perhaps owing to differences in irrigation setups or scheduling across studies. Simple drench applications produced consistently good results, albeit with a somewhat lower average effect than drip. Drenching is a more concentrated, one‐off treatment and its efficacy may depend on factors such as application volume to ensure adequate soil penetration as well as immediate post‐application moisture. Current commercial practice in soft‐fruit crops often favour drip applications, whereas drenching is more common in ornamental crops.^22^ These findings confirm that both methods can achieve vine weevil control, so practical considerations such as cost, labour and compatibility with the grower's existing infrastructure will determine the choice in each case. Regardless of method, adequate post‐application irrigation remains a critical best practice.^29^

The moderator analyses indicate that EPN efficacy against vine weevil is optimised through attention to application timing and environmental context rather than through choice of application method or species alone. Growers can achieve reliable control by applying EPNs when soil temperatures reach 18–30 °C, targeting active larval stages and ensuring adequate soil moisture postapplication. Protected cropping environments offered more consistent outcomes than outdoor applications, which is likely to reflect better environmental stability. Although all five EPN species provided significant larval reductions, the modest differences among them suggest that species selection should be guided primarily by temperature requirements (e.g. *S. kraussei* for cooler conditions) rather than assuming large efficacy differences. Both drench and drip application methods can succeed when these core environmental conditions are met, allowing practical considerations such as cost, labour and existing infrastructure to guide method selection. The practical value of optimising EPN deployment is reinforced by the economic and ecological advantages they offer relative to synthetic chemical insecticides. Unlike synthetic chemical insecticides that require repeated seasonal applications, EPNs can persist in suitable substrates, with field studies documenting effective suppression lasting from several months to over a year depending on species and substrate characteristics.^57^, ^58^ This extended residual activity reduces application frequency and associated labour costs, partially offsetting higher initial product costs compared to synthetic chemical alternatives. EPNs also have minimal nontarget effects on beneficial arthropods including predatory mites and pollinators, in contrast to broad‐spectrum synthetic chemical insecticides that can disrupt integrated pest and pollinator management programmes, and cause secondary pest outbreaks.^59^, ^60^, ^61^ These ecological benefits align with regulatory trends toward restricting problematic insecticides from use in commercial production systems.^1^ Consumer demand for reduced synthetic chemical pesticide residues has created premium markets, with willingness‐to‐pay studies indicating price premiums averaging 29% for crops produced with minimal synthetic chemical inputs.^62^ EPNs can be integrated with other biological control agents including EPFs, although species compatibility requires evaluation as some combinations show synergistic effects while others exhibit antagonism.^63^ Although there are some synthetic chemical insecticide options to control adult vine weevil, EPNs represent a control option that, when deployed under appropriate conditions identified in this meta‐analysis, provides reliable vine weevil larval suppression.

During this review, it became apparent that many published studies on EPN efficacy did not meet basic quality criteria required for inclusion in a quantitative analysis. Although the meta‐analysis included 162 comparisons from 23 independent studies, many others were excluded because of shortcomings in experimental design or reporting. A frequent issue was the lack of an appropriate control, with some studies applying EPNs and reporting mortality without a control group for comparison. Without a control, it is impossible to quantify EPN treatment effect to distinguish natural mortality or other factors from treatment‐induced mortality in a rigorous way. Another pervasive problem was inadequate reporting of variance and sample size. Meta‐analysis requires, at minimum, means and some measure of variance (e.g. SD or SE) for both treatment and control.^64^ However, several vine weevil papers present efficacy data only in qualitative terms or as percentages without any statistical dispersion. Such reports, while perhaps useful directionally, cannot be included in a meta‐analysis. Any comparisons lacking a SD or other variance estimate were excluded from the dataset, because effect sizes cannot be estimated without them.^65^ Laboratory experiments and anecdotal observations also were excluded to avoid unrealistic or unquantifiable data. Many laboratory assays use conditions (e.g. Petri dishes, very high nematode concentrations or artificially optimal settings) that may inflate efficacy relative to field conditions and including them could overestimate real‐world performance.^18^ By focusing on field and semi‐field experiments, this meta‐analysis aimed to draw conclusions relevant to practical vine weevil management. These methodological limitations in the literature help explain why the evidence base for EPNs appears inconsistent. When studies without controls or variance are omitted, the remaining high‐quality studies provide a more coherent picture. Nonetheless, the fact that a substantial number of published experiments had to be excluded from the meta‐analysis indicates a need for improvement in experimental rigor in this field. Key recommendations include including a proper untreated control, reporting the mean outcome and a measure of variability with sample size for each treatment, and using standardised or at least well‐described outcome metrics.^64^ Adopting more uniform protocols or at least reporting sufficient detail would greatly facilitate future syntheses. Formal risk‐of‐bias assessments indicated that excluding lower‐quality comparisons left the overall effect essentially unchanged, and study quality was not associated with effect size, supporting the robustness of the conclusions. Finally, although there was evidence that smaller studies tended to report larger effects, adjusting for potentially missing studies did not change the pooled estimate, suggesting a limited impact of publication bias on the main conclusion.

## 5 CONCLUSION

This meta‐analysis confirms that under nearly all tested conditions EPNs achieve a large reduction in vine weevil larval survival. These findings validate the effectiveness of EPN‐based biological control in field and semi‐field situations, demonstrating that such biological control agents can substantially suppress vine weevil populations under typical crop production conditions. However, considerable variability in efficacy was evident across studies, with the magnitude of control influenced by factors such as crop type (soft fruit *versus* ornamental), cropping environment, application method, growing medium and soil temperature at application. Temperature and protected cropping were the most informative moderators in univariate analyses, although moderator signals attenuated under multilevel CR2‐robust inference. Higher soil temperatures consistently led to greater larval mortality, highlighting temperature as a critical factor for EPN success. Real‐world variability should be expected (prediction interval crossing zero), but the probability of benefit remains high on average. Further research is needed to clarify how these variables interact and to determine optimal deployment strategies for different cultivation contexts. Moreover, the meta‐analysis revealed significant scope to improve the design and reporting of EPN efficacy studies. Although some experiments were conducted to high standards, many others lacked proper controls or failed to report variability, leading to their exclusion from the quantitative synthesis. Risk‐of‐bias sensitivity and moderation indicated that the central efficacy result is robust to plausible biases. Addressing these methodological shortcomings in future research will expand the reliable evidence base and enable more definitive conclusions to guide EPN use in practice.

## Supporting information


**Data S1.** Supporting Information.

**Data S2.** Supporting Information.

## Data Availability

Supporting information File S1 contains the complete dataset of extracted comparisons from 23 studies, including: study identifiers, raw data (mean live larvae, standard deviations, sample sizes for treatment and control groups), moderator variable classifications (EPN species, crop type, cropping context, application method, growing medium, soil temperature categories), and risk‐of‐bias assessments across five domains (randomization, allocation, blinding, outcome measurement, other sources) and an indicator variable (included_in_analysis) identifying which comparisons were retained after applying variance‐based exclusion criteria. File S2 provides the complete annotated R scripts (R v4.4.0) for effect size calculation, meta‐analysis, moderator analyses, sensitivity analyses, publication bias assessment and figure generation.
